# Endoplasmic reticulum stress and unfolded protein response in immune cell function

**DOI:** 10.3389/fimmu.2025.1694102

**Published:** 2025-10-24

**Authors:** Goshi Matsushima, Yuhki Yanase, Tadashi Nakagawa, Mitsuhiro Goda, Koichiro Ozawa, Toru Hosoi

**Affiliations:** ^1^ Department of Clinical Pharmacology and Therapeutics, Graduate School of Biomedical and Health Sciences, Hiroshima University, Hiroshima, Japan; ^2^ Department of Clinical Pharmacology, Faculty of Pharmaceutical Sciences, Sanyo-Onoda City University, Yamaguchi, Japan; ^3^ Faculty of Pharmacy, Yasuda Women’s University, Hiroshima, Japan

**Keywords:** endoplasmic reticulum stress, unfolded protein response, immune cells, PERK (PKR-like endoplasmic reticulum kinase), ATF6 (activating transcription factor 6), IRE1 (inositol-requiring enzyme 1)

## Abstract

Endoplasmic reticulum (ER) stress and the unfolded protein response (UPR) have emerged as central regulators of immune cell function and inflammatory processes. The UPR, mediated by three principal ER-resident sensors, IRE1α, PERK and ATF6, maintains cellular homeostasis under stress conditions but also contributes to pathogenesis when dysregulated. Recent studies revealed that the UPR plays critical roles not only in protein folding but also in directing immune cell fate, activation, and cytokine production. Although significant advances have been made, various questions remain regarding the cell-type-specific and context-dependent functions of ER stress responses. Understanding these mechanisms would be crucial for developing targeted therapies. Therefore, in this review, we provide a comprehensive overview of how ER stress and the UPR influence various immune cell types, including monocytes, macrophages, dendritic cells, granulocytes, T cells, B cells, microglia, and astrocytes, within both peripheral and central immune systems.

## 1 Introduction: ER Stress and UPR

**Figure 1:**
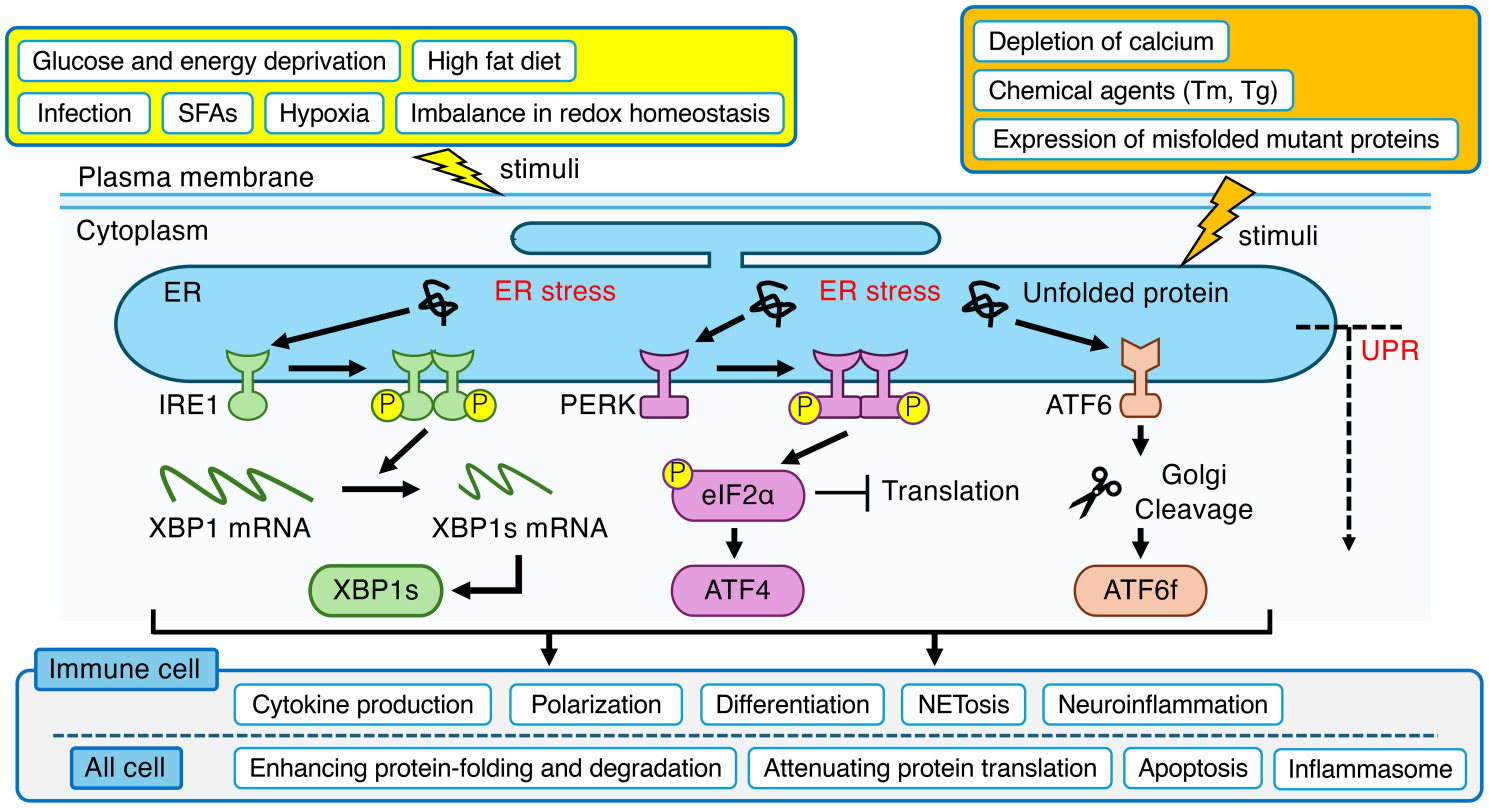
Overview of the ER stress and UPR in mammalian cell. ER stress, triggered by a wide range of physiological and pathological stimuli, leads to the activation of three canonical sensor proteins (IRE1, PERK and ATF6) located on the ER membrane. These pathways orchestrate an adaptive program to restore homeostasis in ER. Beyond this core function, the UPR is intricately linked to the regulation of immune system, modulating critical processes in immune cells such as cytokine production, polarization, differentiation, NETosis, and neuroinflammation. The yellow box indicates indirect stimuli that affect ER function. The orange box indicates direct stimuli that affect ER function. ER, endoplasmic reticulum; UPR, unfolded protein response; IRE1, inositol-requiring enzyme 1; XBP1, X-box-binding protein 1; XBP1s, spliced XBP1; PERK, protein kinase RNA (PKR)-like ER kinase; eIF2α, eukaryotic translation initiation factor-2α; ATF4, activating transcription factor-4; ATF6, activating transcription factor-6; ATF6f, ATF6 fragment; SFAs, saturated fatty acids; Tm, tunicamycin; Tg, thapsigargin; NETs, neutrophil extracellular traps.

Endoplasmic reticulum (ER) is the largest organelle in the mammalian cell, performing a wide range of essential functions, including the synthesis, transport, and folding of proteins (1–3). It is also the primary site for the synthesis of lipids and steroids, carbohydrate metabolism, and calcium storage (1, 4). A dysfunction in the ER’s protein-folding capacity leads to the accumulation of unfolded or misfolded proteins in the ER, a state known as “ER stress” (5). This stress is triggered by a variety of pathological conditions, including depletion of calcium or redox homeostasis, glucose and energy deprivation, hypoxia, the accumulation of misfolded mutant proteins, and pathogen infection (2, 5–8). To counteract this, cells activate a sophisticated signaling network known as the Unfolded Protein Response (UPR). The UPR is orchestrated by three ER-transmembrane sensors: Inositol-requiring enzyme 1 (IRE1), protein kinase RNA (PKR)-like ER kinase (PERK), and activating transcription factor-6 (ATF6) (9) (**Figure 1**). Both IRE1 and ATF6 exist as two isoforms, α and β, and the α-isoforms are considered the primary mediators of the UPR (10, 11). The primary function of IRE1 is to splice X-box-binding protein 1 (XBP1) mRNA, producing a transcription factor, spliced XBP1 (XBP1s), that alleviates ER stress by enhancing protein-folding and degradation pathways (10, 12). PERK mitigates ER stress by phosphorylating eukaryotic translation initiation factor-2α (eIF2α) to globally attenuate protein synthesis, while paradoxically promoting the translation of the activating transcription factor-4 (ATF4), which can induce the key pro-apoptotic factor C/EBP-homologous protein (CHOP) (10, 12). ATF6 responds to ER stress by trafficking to the Golgi, where cleavage liberates its cytosolic domain, ATF6 fragment (ATF6f), to act as a potent transcription factor that primarily upregulates cytoprotective genes (9, 10, 12). Experimentally, these UPR pathways are often studied by inducing ER stress with chemical agents that promote the accumulation of unfolded proteins, most notably tunicamycin (Tm), which inhibits N-linked glycosylation, and thapsigargin (Tg), which disrupts ER calcium homeostasis (13). In its adaptive phase, the UPR aims to restore homeostasis by attenuating protein translation, upregulating ER chaperones, and enhancing ER-associated degradation (ERAD) of misfolded proteins (14). However, under severe and/or prolonged stress, the UPR switches from a pro-survival to a pro-apoptotic program, triggering cell death (15).

Indeed, chronic ER stress and the ensuing dysregulation of the UPR affect the several physical functions of cells, such as secretion of hormones from pancreatic β-cells and adipocytes, resulting in the pathogenesis of numerous human diseases, spanning from metabolic conditions like diabetes and obesity to neurodegenerative disorders such as Alzheimer’s and Parkinson’s disease (16–18). Furthermore, the pathological role of ER stress extends to a range of diseases, including autoimmune conditions like rheumatoid arthritis and systemic lupus erythematosus, as well as cancer (19–23).

A state of chronic and/or low-grade inflammation is now understood to be a key pathological hallmark at the root of a wide range of diseases, including type 2 diabetes, cancer, rheumatoid arthritis and Alzheimer’s disease (24–29). Central to these inflammatory processes are immune cells, whose aberrant activation and dysfunction play key roles in disease progression (30, 31). Within these immune cells, the ER serves as a critical nexus for sensing cellular status and orchestrating adaptive responses. Therefore, ER stress and the UPR may contribute to various diseases by affecting immune cell function and thereby inducing various inflammation.

Currently, the information on the role of ER stress and UPR in immune cells remains insufficient. In this review we focused on and summarized current understanding of the association of ER stress and UPR with immune cell functions.

## 2 ER stress in immune cells as a driver of inflammation

ER stress has emerged as a critical modulator of the development, activation, and effector functions of both innate and adaptive immune cells. Each of these cell types relies on the ER not only for the synthesis and processing of proteins but also for integrating environmental signals that influence their fate and function. Dysregulated ER stress responses can thus profoundly affect immune homeostasis and contribute to pathological inflammation and tissue damage.

### 2.1 Peripheral immune cells

#### 2.1.1 Monocytes

Monocytes circulate in the blood, defend against pathogens via phagocytosis, and differentiate into macrophages or dendritic cells (DCs) to support immune responses and tissue repair (32).

Tg-induced ER stress has been shown to induce an inflammatory phenotype in monocytes, leading to the increased mRNA expression of pro-inflammatory cytokines interleukin-6 (IL-6) and IL-8 (33). Furthermore, Tg-induced ER stress in monocytes has been shown to amplify TNF-α production in response to stressors such as lipopolysaccharide (LPS) and palmitic acid (34).

#### 2.1.2 Macrophages

Macrophages are a diverse population of innate immune phagocytes found in all tissues, where they act as sentinels essential for homeostasis, tissue repair, and host defense (35). Conventionally, their activation states are described as a spectrum between two main poles: the pro-inflammatory ‘M1’ phenotype, which is central to host defense against infection, and the anti-inflammatory, tissue-reparative ‘M2’ phenotype (36).

In macrophages, saturated fatty acids (SFAs) engage the IRE1α pathway to promote the activation of the nucleotide-binding oligomerization domain-like receptor family, pyrin domain-containing 3 (NLRP3) inflammasome, which in turn leads to the secretion of IL-1β, a pro-inflammatory cytokine closely linked to insulin resistance (37). A key study by Shan et al. demonstrated that myeloid-specific deletion of IRE1α protects mice from diet-induced obesity and insulin resistance by promoting a shift from pro-inflammatory M1 to anti-inflammatory M2 macrophage polarization in adipose tissue (38). Corroborating the therapeutic importance of these genetic findings, pharmacological inhibition of IRE1α has also been shown to be highly effective. In mice with diet-induced obesity, administering a specific inhibitor of IRE1α’s RNase activity (STF-083010) significantly ameliorated insulin resistance and protected against obesity by increasing thermogenesis (39). The primary mechanism involved reducing the accumulation of pro-inflammatory adipose tissue macrophages (ATMs), specifically the ‘M1-like’ CD11c^+^ and metabolically activated CD9^+^ subsets, thereby curtailing adipose inflammation (39). While the IRE1α pathway is a clear driver of this process, other branches of the UPR also contribute to the activation of macrophages, including the PERK-ATF4 axis. Indeed, a study demonstrated that deficiency of ATF4, a transcription factor downstream of the PERK pathway, suppresses the SFA-induced expression of the pro-inflammatory cytokine IL-6 in macrophages (40). In addition to these macrophage-intrinsic pathways, a key study demonstrated that in obese mice, high fat diet-induced CHOP expression, particularly within adipocytes, alters the local tissue environment in a way that drives the polarization of ATMs towards the pro-inflammatory M1 phenotype, resulting in the induction of insulin resistance (41).

#### 2.1.3 Dendritic cells

As professional antigen-presenting cells, DCs play critical roles for innate and adaptive immune systems (42). DCs orchestrate the adaptive immune response by presenting captured antigens to naive T cells to provide activation signals, and by producing key instructive cytokines, such as IL-12 and IL-23, for direct T cell differentiation (42–44).

Although dispensable for DCs homeostasis in the steady state, upon activation by both R848 and palmitic acid, ATF6α contributes to the production of critical pro-inflammatory cytokines, including IL-12p70 and IL-6 (45). Beyond the ATF6α pathway, other arms of the UPR also act as potent modulators of cytokine production in activated DCs. It has been demonstrated that inducing ER stress with classical chemical stressors, such as Tm or Tg, in DCs stimulated with pathogen-associated molecular patterns (PAMPs) markedly enhanced the mRNA expression of the pro-inflammatory cytokine IL-23 (46). The underlying mechanism was shown to be dependent on specific UPR branches; the IRE1α pathway was essential for the IL-23 response to the fungal PAMP zymosan, while the PERK pathway was required for the response to the bacterial PAMP LPS. In addition, XBP1s has been shown to be indispensable for the development and survival of DCs (47). Recent reports indicate that tripartite motif containing 29 (TRIM29), known as a member of E3 ubiquitin ligase, promotes PERK-mediated ER stress immune response by inducing SUMOylation and stability of PERK (48). Moreover, TRIM29 is reported to negatively regulate the innate immune response against virus infections by inhibiting production of type I IFNs, such as IFN-α and IFN-β, in DCs and macrophages (49–51). These findings suggest that TRIM29-PERK axis would be a target for ER stress-associated immune disorders.

#### 2.1.4 Granulocytes (neutrophils, eosinophils and basophils)

Granulocytes, which include neutrophils, eosinophils, and basophils, play important roles in inflammation, encompassing both pathogen clearance and immunoregulation (52).

Neutrophils are the most abundant leukocytes and essential first responders in acute inflammation, where they contribute to host defense and tissue repair (53). However, they exacerbate disease through mechanisms including the release of proteases, such as neutrophil elastase, and the formation of Neutrophil Extracellular Traps (NETs), which has established them as a promising therapeutic target for a range of chronic inflammatory conditions (53).

In lupus hyperactivated IRE1α in neutrophils directly drives pathological NETosis, a highly inflammatory process, as demonstrated by the finding that pharmacological inhibition of IRE1α (4μ8C) reduces this process (54).

Eosinophils are now understood to be versatile immunomodulatory cells that bridge innate and adaptive immunity (55). They fulfill this role by modulating the functions of B and T cells and through antigen presentation. Furthermore, they communicate extensively with other innate immune cells, including macrophages and DCs, to regulate the overall inflammatory environment. A study revealed that the IRE1α-XBP1s pathway is selectively and absolutely required for eosinophil differentiation, while being dispensable for the development of other granulocytes like neutrophils (56).

Basophils, rare granulocytes sharing features of both innate and adaptive immunity, contribute to allergic inflammation by expressing the high-affinity IgE receptor (FcϵRI) and releasing mediators, such as histamine, in response to IgE-mediated stimulation (57, 58). The role of ER stress in basophils remains poorly defined, though initial evidence suggests a distinct reliance on the UPR. IgE receptor-mediated stimulation of basophils leads to the activation of the IRE1α pathway (59). However, the physiological significance of this activation is not yet well understood.

#### 2.1.5 T cells

T cells play a pivotal role in directing the adaptive immune response, which ensures the effective and specific clearance of invading pathogens (60). Conversely, dysfunction of T cell development or activity contributes to the pathogenesis of a wide spectrum of human illnesses, such as immunodeficiencies, autoimmune conditions, and allergic disorders. Conventional T cells are broadly divided into two main classes: CD4^+^ helper T cells that orchestrate the immune response, and CD8^+^ cytotoxic T cells that eliminate target cells (61).

In tumor-infiltrating T cells, the PERK pathway of the UPR has been identified as a key driver of cellular exhaustion and energy depletion (62). Pharmacological (GSK2606414) or genetic inhibition of the PERK pathway in T cells was shown to preserve their energy reserves and enhance their anti-tumor effector functions, suggesting that targeting ER stress is a promising strategy to bolster T cell-mediated immunity (62). UPR also plays a critical role in shaping the differentiation of specific T helper cell subsets. This is particularly evident in the case of T helper-17 (Th17) cells, a pro-inflammatory subset strongly implicated in the pathogenesis of autoimmune diseases. A key study revealed that ER stress inducers like Tm and Tg, markedly enhance the differentiation of naive T cells into Th17 cells (63). The IRE1α-XBP1s pathway is also crucial for the function of T helper 2 (Th2) cells, a subset involved in allergic responses and anti-helminth immunity. The IRE1α-XBP1s pathway plays a critical role during Th2 cell activation by regulating the expression and secretion of their signature cytokines, as well as their proliferation (64). ER stress also critically influences regulatory T cells (Tregs), which are essential for immune tolerance and inflammation control. ER stress has been shown to mediate the detrimental effects of the stress hormone cortisol on Tregs function (65). Specifically, cortisol exposure in the presence of a specific antigen reduces TGF-β expression from Tregs, an effect that is prevented by the ER stress inhibitor BiP inducer X. Beyond this, ER stress also plays critical roles in the broader dysregulation of Th17/Treg balance (66). For example, the ER stress inhibitor, 4-phenylbutyric acid (4-PBA), has been demonstrated to suppress reactive oxygen species (ROS) production, consequently inhibiting Th17 differentiation and promoting Treg differentiation. In addition, it has been reported that in patients with ulcerative colitis, a synergistic effect between environmental factors and ER stress inhibits the differentiation of Tr1 cells, an IL-10-producing subset of Tregs (67).

#### 2.1.6 B cells and plasma cells

B cells perform a variety of crucial immune functions, including not only their classical roles in antibody production and antigen capture via the B cell receptor (BCR), but also their capacity to act as antigen-presenting cells (68, 69).

The differentiation of B cells into professional antibody-secreting plasma cells is a process critically dependent on the IRE1α-XBP1s pathway (70, 71). These plasma cells can produce a diverse repertoire of antibody classes, each tailored for distinct and specialized effector functions within the immune system (68). While the IRE1α pathway is critical for differentiation, it is also co-opted by malignant plasma cells in multiple myeloma, where its inhibition suppresses the secretion of not only immunoglobulin light chains but also key growth factors and cytokines, such as vascular endothelial growth factor (VEGF), IL-1α, IL-6, and IL-8 (72).

### 2.2 Neuronal cells

#### 2.2.1 Microglias

Microglia are the resident immune cells of the central nervous system, forming a dynamic and motile network that continuously surveys their local environment (73).

ER stress in microglia has been shown to drive pathological inflammatory responses, while its pharmacological inhibition with 4-PBA is protective (74). Furthermore, a key study utilized mice with a microglia-specific deletion of the ER stress sensor IRE1α. When challenged with a high fat diet, male mice with this deletion were significantly protected from obesity, glucose intolerance, and hypothalamic inflammation compared to their wild-type counterparts (75). In addition to the IRE1α pathway, the PERK branch of the UPR has also been implicated as a key driver of pro-inflammatory microglial polarization. For instance, a study using LPS-stimulated microglia showed that the compound ascorbic acid 6-palmitate significantly inhibited the activation of the PERK/eIF2α pathway (76). This suppression of ER stress, in turn, helped restore the M1/M2 balance by promoting an anti-inflammatory M2 phenotype, evidenced by increased expression of IL-10 and Arginase-1. High glucose, a key feature of diabetes, has also been shown to induce a state of PERK branch activation in microglia. For example, an *in vitro* study demonstrated that exposing microglial cells to hyperglycemic conditions led to the upregulation of the key ER stress markers CHOP and phosphorylated eIF2α via PERK pathway (77). This activation of the pro-apoptotic ER stress pathway highlighted the vulnerability of microglia to glucotoxicity-induced ER stress.

#### 2.2.2 Astrocytes

Astrocytes are the most abundant glial cells in the central nervous system and are essential for neuronal homeostasis and function (78). Although astrocytes are not classically categorized as immune cells due to their neuroepithelial origin, they are increasingly recognized as key regulators and effectors of neuroinflammatory responses (79, 80).

It has been demonstrated *in vitro* that inducing ER stress in astrocytes drives the expression of the pro-inflammatory cytokines, TNF-α and IL-6 (81). Crucially, this inflammatory response was significantly suppressed by co-treatment with a specific PERK inhibitor (GSK2606414), providing direct evidence for the PERK pathway’s causal role in driving astrocyte-mediated inflammation. Beyond increasing cytokine production, PERK activation drives astrocytes into a pathological ‘reactivity state,’ causing them to lose neuroprotective functions while gaining neurotoxic properties. Crucially, a landmark study confirmed this causal link, as astrocyte-specific genetic inhibition of the PERK pathway was sufficient to prevent neuronal loss and extend survival in a prion disease model (82). Furthermore, it has been reported that hyperglycemic conditions activate UPR pathways in astrocytes, resulting in an increased secretion of the pro-inflammatory cytokines TNF-α, IL-6 and IL-18 (83).

### 2.3 ER stress and inflammasome

Inflammasomes function as cytoplasmic platforms that sense PAMPs and/or danger-associated molecular patterns (DAMPs), playing a key role in orchestrating host immune homeostasis in various cells (84–86). Upon activation, they recruit and activate caspase-1, which in turn processes pro-inflammatory cytokines such as IL-1β and IL-18 and induces pyroptosis, a lytic form of programmed cell death (84, 85, 87). Among several types of inflammasomes, the NLRP3 inflammasome is the most extensively studied and appears particularly responsive to cellular stress signals (84). Recent studies have elucidated that ER stress-induced UPR activates NF-κB signaling, leading to promotion of expression of NLRP3 and IL-1β, resulting in activation of inflammasome (88). Moreover, ER stress-induced calcium leakage from the ER into the mitochondria leads to an increase in mitochondrial ROS (mROS) production. These changes act as triggers for various endocrine system diseases. Indeed, in models of fatty liver ischemia/reperfusion, ER stress in macrophages induces mitochondrial calcium overload, which in turn promotes mROS production and activates NLRP3 signaling (89). In the hyperglycemic state of diabetes, elevated ER stress leads to an increase in NLRP3 inflammasome-dependent IL-1β secretion, which causes β-cell dysfunction and promotes obesity and insulin resistance (90). Similar ER stress-inflammasome pathways have been implicated in neurodegenerative disease. In Parkinson’s disease, pathological α-synuclein aggregates have been shown to induce a profound ER stress response in microglia, which subsequently promotes the activation of the NLRP3 inflammasome (91). Thus, ER stress also contributes to the activation of inflammasome which plays central roles in the pathogenesis of chronic inflammation, metabolic diseases, and neurodegenerative conditions.

### 2.4 Relation of pro-inflammatory cytokines released from immune cells to endocrine cells

The release of pro-inflammatory cytokines from immune cells is a critical driver of widespread endocrine dysfunction. Cytokines, such as TNF-α and IL-6, have been shown to directly impair insulin receptor signaling in metabolic tissues, thereby contributing to the development of insulin resistance (92–94). Moreover, pro-inflammatory cytokines, including IL-1, IL-6, and TNF-α, are implicated in glucocorticoid resistance, thereby compromising the body’s endogenous anti-inflammatory feedback mechanisms (95, 96). In addition to interfering with hormone signaling, pro-inflammatory cytokines induce ER stress in endocrine tissues, resulting in disruptions in protein folding, hormone synthesis, and secretion, and may ultimately promote apoptosis (6). Notably, in pancreatic β-cells, pro-inflammatory cytokines, IL-1β and IFN-γ, amplify ER stress, resulting in the pathogenesis of type 1 diabetes (97). Additionally, IFN-α induces ER stress in thyroid cells, which lead to thyroid cell apoptosis (98).

## 3 Conclusion

ER stress and the UPR are now recognized as central regulators of immune cell fate and function. Far beyond their canonical roles in protein quality control, the UPR branches, IRE1α, PERK and ATF6, serve as critical signaling hubs that translate environmental and metabolic cues into immune responses. This review highlighted how ER stress shapes inflammation by modulating cytokine production, cell differentiation, and polarization across diverse immune cell types, including monocytes, macrophages, DCs, granulocytes, T cells, B cells, and glial cells such as microglia and astrocytes (**Figure 1**; **Table 1**). Notably, dysregulated ER stress skews immune responses toward pathological inflammation, contributing to the progression of metabolic, autoimmune, neurodegenerative, and malignant diseases. However, our understanding remains incomplete. Various questions remain regarding cell-type specificity, temporal dynamics, and the crosstalk between UPR pathways. Future studies should aim to unravel these complexities using conditional genetic models and systems-level approaches. Moreover, targeted pharmacological modulation of specific UPR branches holds therapeutic promise for controlling inflammation without compromising essential ER functions. In conclusion, deciphering the immunological roles of ER stress responses offers a novel and fertile avenue for therapeutic intervention in a wide range of inflammatory diseases.

**Table 1:** Summary of the roles of the UPR in immune cells.

Cell	UPR pathway /molecule	Function	Reference
Monocyte	ER stress	IL-6 and IL-8 production. Enhancement of LPS- and palmitic-induced TNF-α production.	(33) (34)
Macrophage	IRE1α	IL-1β secretion by SFAs. M1 polarization. Accumulation of pro-inflammatory ATMs.	(37) (38) (39)
	ATF4	SFA-induced expression of IL-6.	(40)
Adipocyte	CHOP	Polarization of macrophages towards the M1 phenotype.	(41)
DCs	ATF6α	Production of IL-12p70 and IL-6.	(45)
	IRE1α	Enhancing IL-23 expression by fungal PAMP zymosan.	(46)
	PERK	Enhancing IL-23 expression by bacterial PAMP LPS.	(46)
	XBP1	Development and survival.	(47)
Macrophage/DCs	PERK	Suppression of type I IFNs production.	(49–51)
Neutrophil	IRE1α	NET formation.	(54)
Eosinophil	IRE1α-XBP1s	Differentiation.	(56)
Basophil	IRE1α	Activated by IgE receptor stimulation.	(59)
T cell	PERK	Cellular exhaustion and energy depletion (tumor-infiltrating T cells). Attenuation of anti-tumor effector functions.	(62)
	ER stress	Differentiation of naive T cells into Th17 cells.	(63)
	IRE1α-XBP1s	Activation, regulating their cytokine expression, secretion, and proliferation of Th2 cell.	(64)
	ER stress	Decreased TGF-β expression from Treg.	(65)
	ER stress	Promoting Th17 differentiation.	(66)
	ER stress	Inhibition of Tr1 cell differentiation.	(67)
B cell/Plasma cell	IRE1α-XBP1s	Differentiation into plasma cells.	(70, 71)
	IRE1α	Secretion of immunoglobulin light chains, VEGF, IL-1α, IL-6, and IL-8.	(72)
Microglia	ER stress	Neuroinflammation.	(74)
	IRE1α	Enhancement of high fat diet-induced obesity, glucose intolerance, and hypothalamic inflammation.	(75)
	PERK/eIF2α	Suppression of promotion to M2 type.	(76)
	p-eIF2α/CHOP	Activated by high glucose.	(77)
Astrocyte	PERK	Expression of TNF-α and IL-6. Neuronal loss.	(81) (82)
	ER stress	Enhancement of high glucose-induced IL-6, TNF-α and IL-18 secretion.	(83)
